# The role of lactylation in breast cancer development: mechanisms, clinical translation and new strategies for treatment

**DOI:** 10.3389/fonc.2025.1665097

**Published:** 2025-11-05

**Authors:** Yanzhen Lu, Xiaoting Yang, Lulu Tan, Yunfei Yang, Dan Yu, Gang Feng, Yuyan Tan

**Affiliations:** ^1^ Department of Thyroid and Breast Surgery, The First College of Clinical Medical Science, China Three Gorges University & Yichang Central People’s Hospital, Yichang, China; ^2^ China Three Gorges University, Yichang, China

**Keywords:** breast cancer, lactate, lactylation, targeted therapy, tumor microenvironment

## Abstract

Worldwide, breast cancer (BC) is a common and deadly illness that poses a serious risk to women’s health. Its development is intimately associated with tumor microenvironment (TME) alteration and metabolic problems. Lactic acid, a principal byproduct of glycolysis, not only facilitates the acidity of the TME but also interferes with cellular circadian rhythms. Moreover, it exerts multifaceted regulatory effects on breast cancer growth by facilitating a new post-translational modification(PTM)ficatio lactylation (Kla). By accelerating metabolic reprogramming, encouraging immunological microenvironment dysregulation, and intensifying tumor growth, metastasis, and chemoresistance, Kla has been shown in studies to contribute to the advancement of BC and poor prognosis. Lactate production and transport, especially targeting lactate dehydrogenase (LDH) and monocarboxylate transporter protein (MCT), show promise in BC treatment. Both tumor-suppressive and immunotherapy-enhancing effects are exhibited by inhibitors that target LDH and MCTs, and they may work in concert with immunotherapy. The function of Kla in BC, its underlying processes, and the possibility of treating the condition by specifically targeting Kla are all examined in this review. Additionally, it suggests the creation of precision-targeted treatments, providing fresh viewpoints on metabolic treatments and combination treatments for BC.

## 1 Introduction

One of the most prevalent malignant tumors affecting women globally, BC has become more widespread in recent years (1). While much research has been done on diagnosing and treating BC, little is known about the molecular pathways behind its onset and progression. Lactate, which is present in practically all cell types, is produced by glycolysis. Lactate is today recognized as an essential chemical that links cellular metabolism to the regulation of cellular function, despite its initial classification as merely an energy substrate and metabolic waste (2). LDH breaks down pyruvate, which is the primary mechanism that generates lactate. It can either undergo reversible oxidation to pyruvate in the mitochondria to participate in the tricarboxylic acid cycle (TCA cycle) or undergo gluconeogenesis to return to glucose. Normally, intracellular glucose undergoes glycolysis and oxidative phosphorylation to produce large amounts of ATP. However, in order to adapt to the hypoxic, acidic, and nutrient-deficient TME, tumor cells preferentially use glycolysis to make energy, even in the presence of oxygen, in contrast to healthy cells. The Warburg effect is the term for this (3). This effect is due to the increased uptake of glucose and the production of lactate by cancer cells, regardless of the oxygen availability (4). In recent years, advances in proteomics have revealed an important novel epigenetic regulatory mechanism in cancer, namely lactate-mediated protein Kla (5). This discovery opens up a new line of enquiry into tumor biology in general and lactate metabolism in particular.

In 2019, Zhang’s team was the first to publish on histone Kla. They discovered that lactate functions as a precursor to Kla and that both bacteria and hypoxia promote lactate synthesis and glycolysis. Specifically, the writing enzyme p300 enabled H3K18la to enhance the transcription of genes associated with the M2-type phenotype in M1-type macrophages, such as Arg1 (6). Histone Kla is one important epigenetic modification that has a dose-dependent effect on target gene transcription depending on lactate levels. Many studies have demonstrated that Histone Kla plays a role in the development of tumors (7, 8). By functioning as a signaling molecule, lactate can also alter the TME. Lactic acid generated by tumor cells can either promote immunosuppressive cells or, by releasing them, prevent immune cells from preventing tumors, so facilitating immunological escape (9). Interestingly, Sun et al. (10) found that hypoxia in cancer-associated fibroblasts (CAFs) promotes the production of lactic acid, which facilitates the proliferation of BC cells. It is interesting to note that studies have shown that protein Kla is a dynamic and reversible process (11), suggesting that it may be a promising therapeutic target for cancer treatment (12). Developing new oncological treatment strategies may be aided by a comprehensive understanding of the part Kla plays in the onset and progression of cancer. This would broaden our understanding of lactate metabolism and offer new insights and molecular pathways for future medication discovery.

LDH is the primary enzyme that catalyzes the reversible conversion of pyruvate to lactate, which is the last stage of glycolysis. The LDH enzyme is made up of two subunits, “A” (also called the M-type) and “B” (sometimes called the H-type), which combine to generate five different isoenzymes (13). The liver and skeletal muscle contain the majority of LDH-A, also known as the “M” subunit, while the heart muscle has the majority of LDH-B, also known as the “H” subunit. The former is mostly found in the cytoplasm, mitochondria, and other organelles, whereas the latter is mostly found in the mitochondria (14). There is at least some overlap in the functions of LDH-A and LDH-B, and one subunit can partially replace the other. In isolated mitochondria, suppression of LDH results in decreased mitochondrial metabolism when organelles are cultured in lactate instead of pyruvate (15). In cells with active glycolysis, LDH-5 (which consists of four A subunits) predominates because it has the highest affinity for pyruvate and favors its conversion to lactate (16, 17). In tumor metabolism, LDH serves as the central executor of the Warburg effect, converting pyruvate into lactate to drive cancer cells’ reliance on glycolysis for energy. Although this metabolic pathway is less efficient in energy production, it facilitates rapid synthesis of biomolecules to meet the sustained proliferation demands of tumor cells (18). LDH-mediated significant lactate buildup acidifies the tumor microenvironment, which inhibits immune cell function, encourages tumor invasion and metastasis, and activates stromal cells to provide a milieu that supports tumor growth (18). In breast cancer, LDH-A expression is significantly upregulated, with elevated mRNA and protein levels correlating closely with poor patient prognosis (19). Beyond metabolic processes, LDH-A plays a complex role in the development and progression of breast cancer by directly promoting tumor growth by triggering signaling pathways such RAC1 GTPase (20). In conclusion, LDH is more than just a metabolic enzyme. It is inherently connected to the growth of tumors and acts as a key regulator of energy metabolism in breast cancer cells. As such, it is a prospective therapeutic target as well as a useful disease monitoring indicator.

By integrating recent studies in this rapidly evolving field, this review aims to provide a comprehensive knowledge of the complex relationship among immune responses, metabolic reprogramming, circadian rhythm, and the therapeutic effects of lactate and Kla in BC. This all-encompassing approach may yield valuable data for developing innovative therapy modalities that could improve patient outcomes.

## 2 Molecular mechanisms of lactylation

**Figure 1:**
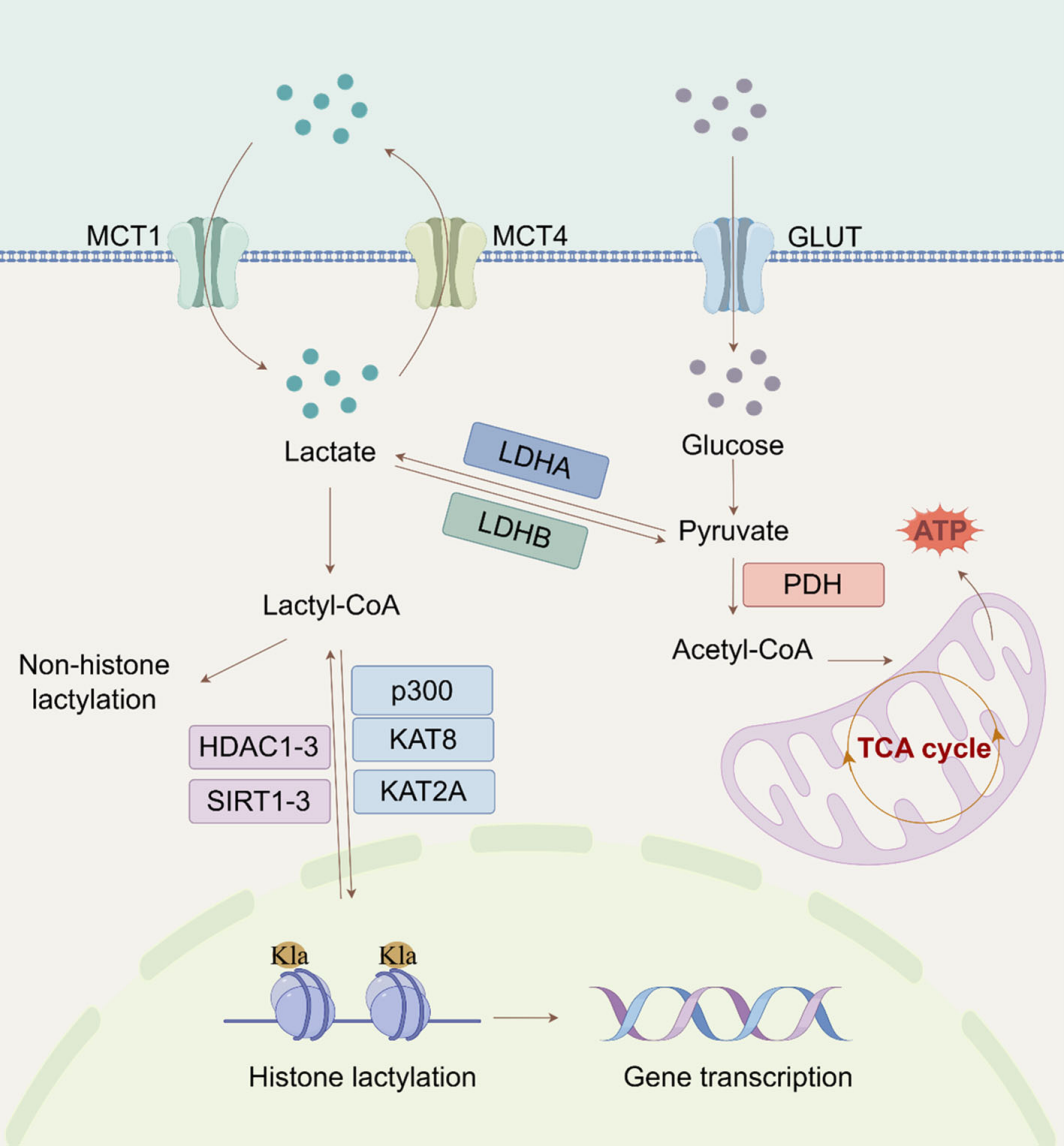
Regulatory mechanisms of lactylation (By Figdraw). The extracellular lactate enters the cytoplasm through MCT and is converted into pyruvate. Subsequently, pyruvate can enter the mitochondria to participate in the tricarboxylic acid cycle. The metabolic process of lactic acid also involves LDH, which converts pyruvate back into lactate. Lactate can also affect histone (H3K18/14la) lactylation through acetyl-CoA, thereby regulating gene expression. (MCT, monocarboxylate transporter protein; Glut, glucose transporters; LDH, lactate dehydrogenase; tricarboxylic acid; PDH, pyruvate dehydrogenase; Kla, lysine lactylation; TCA, cycle tricarboxylic acid cycle; Acetyl-CoA, acetyl coenzyme A).

The discovery of histone Kla demonstrated that Kla can stimulate gene expression through chromatin modification (6). The lysine residues of H2A, H2B, H3, and H4 are the main locations for histone Kla, with H3K18 being a particularly noticeable location (21). Key proteins control the Kla process, which has a complicated mechanism including both enzymatic and non-enzymatic pathways (**Figure 1**). Since then, more studies in this area have shown that Kla can be found on non-histone proteins in addition to histones. Research has demonstrated that both histone and non-histone Kla influence the expression of specific proteins, including NEDD4 and PD-L1 (22). Thus, clarifying the larger epigenetic regulatory network that connects histone and non-histone Kla should be the main goal of future studies.

### 2.1 Enzymatic and non-enzymatic pathways of lactylation

The primary substrate in the enzymatic process is lactyl-CoA (23). CoA-transferases catalyse Kla once lactate is transformed into lactyl-CoA (6). Recent studies using liquid chromatography-tandem mass spectrometry (LC-HRMS) have confirmed the presence of lactyl-CoA in mammalian cells and tissues (24).In the non-enzymatic route, methylglyoxal, a byproduct of glycolysis, conjugates with glutathione by glyoxalase 1 (GLO1) to generate lactosylglutathione (LGSH). Glyoxalase 2 (GLO2) subsequently hydrolyzes LGSH, releasing non-enzymatic acyltransferases that facilitate Kla (9). Trujillo et al. demonstrated that lactate spontaneously transferred from LGSH to CoA via S-to-S acyl transfer results in lactoyl-CoA, which in turn induces histone lysine residues to be D- Kla (25). Furthermore, a different mechanism was suggested by Zong et al. (26) in which lactate is directly transferred to lysine residues through alanyl-tRNA synthetase 1 (AARS1), resulting in L-Kla. Remarkably, lactate-induced and LGSH-associated Kla have distinct physiologic effects. Lactate-induced Kla promotes macrophage polarization toward an anti-inflammatory phenotype, whereas LGSH-associated Kla is linked to increased production of inflammatory cytokines in macrophages. These findings suggest that Kla’s impact on cellular processes depends on the exact underlying mechanism (6, 23). Further studies are needed to fully comprehend the regulatory networks governing lactate-induced and LGSH-associated Kla in cells.

### 2.2 Key regulatory proteins for lactylation

Protein Kla is a reversible and evolutionarily conserved class of PTMs that is dynamically regulated by specific enzymes or enzyme complexes (27). ‘Writers’ speed up the insertion of acyl groups into target proteins, whereas ‘Erasers’ remove these changes. “Readers” are proteins that can recognize these alterations and bind to them to mediate further effects (28, 29). CBP/p300 is the first known writer of histone Kla because lactyl-CoA binds to it powerfully and specifically to induce histone Kla (30). Another major lactyltransferase that has been found is KAT8, which is especially crucial for catalyzing the addition of lactyl groups to protein substrates. Tumorigenesis is one of the biological processes in which this modification is essential. KAT8 facilitates Kla of the eukaryotic translation elongation factor eEF1A2 at lysine 408, which advances colorectal cancer (CRC) (31). Additionally, new studies have shown that KAT2A acts as a lactyltransferase when connected to ACSS2, resulting in Kla at H3K14 and H3K18 (32). To completely comprehend the biological role of KAT2A, a newly identified lactyltransferase, more investigation is needed. There are known Kla ‘Erasers’, including class I histone deacetylases (HDAC1-3), mitochondrial AARS2, and sirtuins (SIRT1-3) (33, 34). The primary *in vivo* deacetylase, SIRT2, is 1000 times less active than HDAC3 (35). SIRT2 has strong deacetylase activity for synthetic peptides connected to PKM2 and efficiently removes Kla at many histone sites *in vitro* and in neuroblastoma cells (36). However, because of its mainly cytoplasmic position, SIRT2 may have a significant influence on the D-Kla of cytoplasmic proteins (35). There is a paucity of research on ‘Readers’ who specialize in Kla. Hu et al.’s (37) proteomic research provided the first proof that BRG1 binds to H3K18la and demonstrates its role as a Kla reader. It also showed that BRG1 reads H3K18la during induced pluripotent stem cell reprogramming.

## 3 Metabolism of lactate and lactylation in cancer

**Figure 2:**
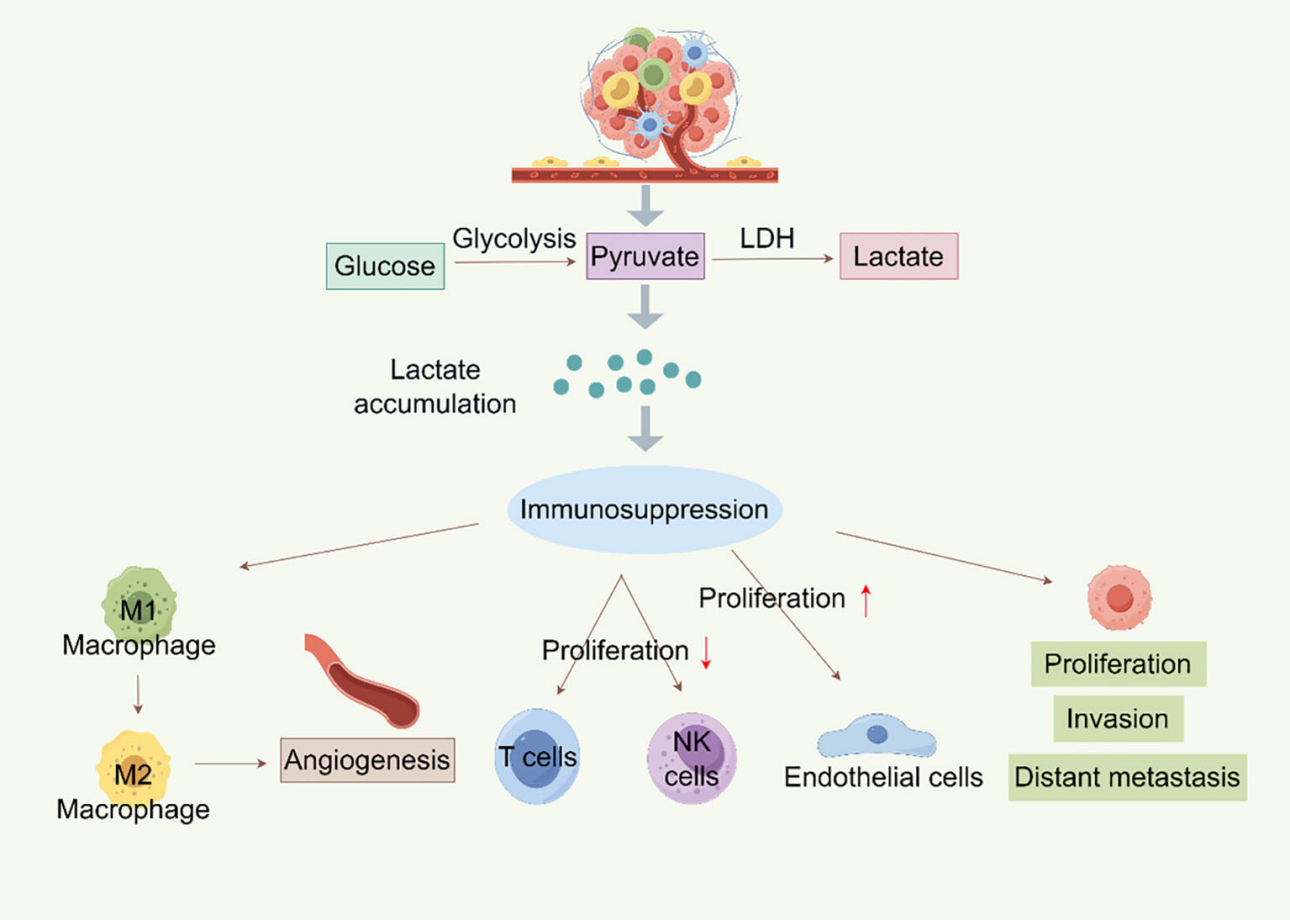
Metabolism of lactylation in tumors (By Figdraw). Lactate is created by glycolysis in tumor cells, and its buildup encourages the growth, invasion, and distant metastasis of tumor cells. Lactate can also cause immunosuppression, which includes NK and T cell proliferation inhibition and M1 macrophage polarization to M2 macrophages, which promotes angiogenesis and endothelial cell growth. Furthermore, LDH can further participate in the glycolysis process by converting lactate into pyruvate, resulting in a vicious loop that encourages tumor growth and immune evasion.

One illustration of how cancer cells’ metabolism differs greatly from that of healthy cells is the Warburg effect. Because of the Warburg effect or other metabolic alterations, lactate, an essential substrate for the protein Kla, accumulates in large quantities in tumors. This accumulation sets off several molecularly mediated pathways that regulate Kla. The lipid bilayer of the cell membrane contains a class of transmembrane proteins known as MCTs, which are responsible for the transport of lactate between intracellular and extracellular compartments (38). There are currently fourteen known MCT isoforms (39). Tumor-oxidizing cells have high levels of MCT1, which primarily mediates lactate uptake, enabling these oxidative cells to use lactate from the TME as an energy source (40). Lactate can enter cancer cells through the MCT1-mediated intercellular shuttle or non-channel pathways (9). Aerobic glycolysis in tumor cells produces lactate, which exacerbates hypoxia, promotes tumor growth, raises lactate production, and stimulates angiogenesis. These processes include invasion, metastasis, and treatment resistance (41, 42) (**Figure 2**). While the Warburg effect is a hallmark of cancer metabolism, recent studies have identified other metabolic features, including metabolic symbiosis, glutamine metabolism, and the “reverse Warburg effect,” wherein CAFs in the tumor stroma cause autophagy and glycolysis to produce lactate (43–46). Additionally, Yu et al. (47) developed two metabolic tags to predict the metabolic phenotypes of cancer cells: one for glycolysis based on gene expression downstream of HIF-1 and one for oxidative phosphorylation (OXPHOS) based on genes downstream of AMPK. Thanks to advancements in metabolic signature analysis, it is now possible to identify metabolic heterogeneity in malignant cells. Xiao et al. (48) used bulk and single-cell RNA sequencing to show that OXPHOS activity variation is a key cause of metabolic heterogeneity in head and neck squamous cell carcinoma and melanoma. Therefore, a theoretical basis for identifying the mechanisms of malignant progression in BC and for creating individualized treatment plans that target metabolic reprogramming will be provided by a thorough resolution of tumor metabolic heterogeneity and the Kla modification networks that are linked to it.

## 4 The regulatory role of Lactylation in breast cancer biology

### 4.1 Lactylation promotes breast cancer tumorigenesis

Kla plays an important role in tumorigenesis. Several investigations have demonstrated that lactate mediates chemoresistance, promotes tumor cell proliferation, and facilitates the formation of the TME (49, 50). A work by Pandkar et al. (51) revealed for the first time the importance of H3K18la in regulating oncogenes associated with the progression of BC, demonstrating that blocking aerobic glycolysis to block the c-MYC–SRSF10 axis decreased the proliferation of BC cells. Furthermore, Hou et al. (52) demonstrated that potassium two pore domain channel subfamily K member 1(KCNK1) promotes histone Kla by binding to and activating LDHA, which in turn increases glycolysis and lactate generation in BC cells. In the end, this encourages the growth, invasion, and metastasis of BC. In a similar vein, Xu et al. (53) discovered that H3K18la increased PPARD expression, which in turn triggered the PI3K/AKT signaling pathway and preserved BC cell viability and proliferation. It’s interesting to note that some research has discovered that triple-negative BC also exhibits substantial lactate-induced protein changes. Specifically, H4K12la is positively connected with the malignant degree of triple-negative breast cancer (TNBC). These discoveries offer fresh perspectives on the processes that underlie the onset and spread of TNBC and could help create efficient treatment plans that target important enzymes that control tumor metabolism or facilitate target gene transcriptional regulation (54). When combined, the research shows that Kla uses a variety of mechanisms to support the advancement of BC. To provide a theoretical basis for the development of precision treatment strategies based on the regulation of TME, it will be necessary to further analyze the spatiotemporal dynamic regulatory network of Kla and confirm the therapeutic potential of targeting Kla metabolic enzymes (like LDHA) or epigenetic effectors through preclinical models.

### 4.2 Lactylation-mediated chemoresistance and DNA damage repair in breast cancer

**Figure 3:**
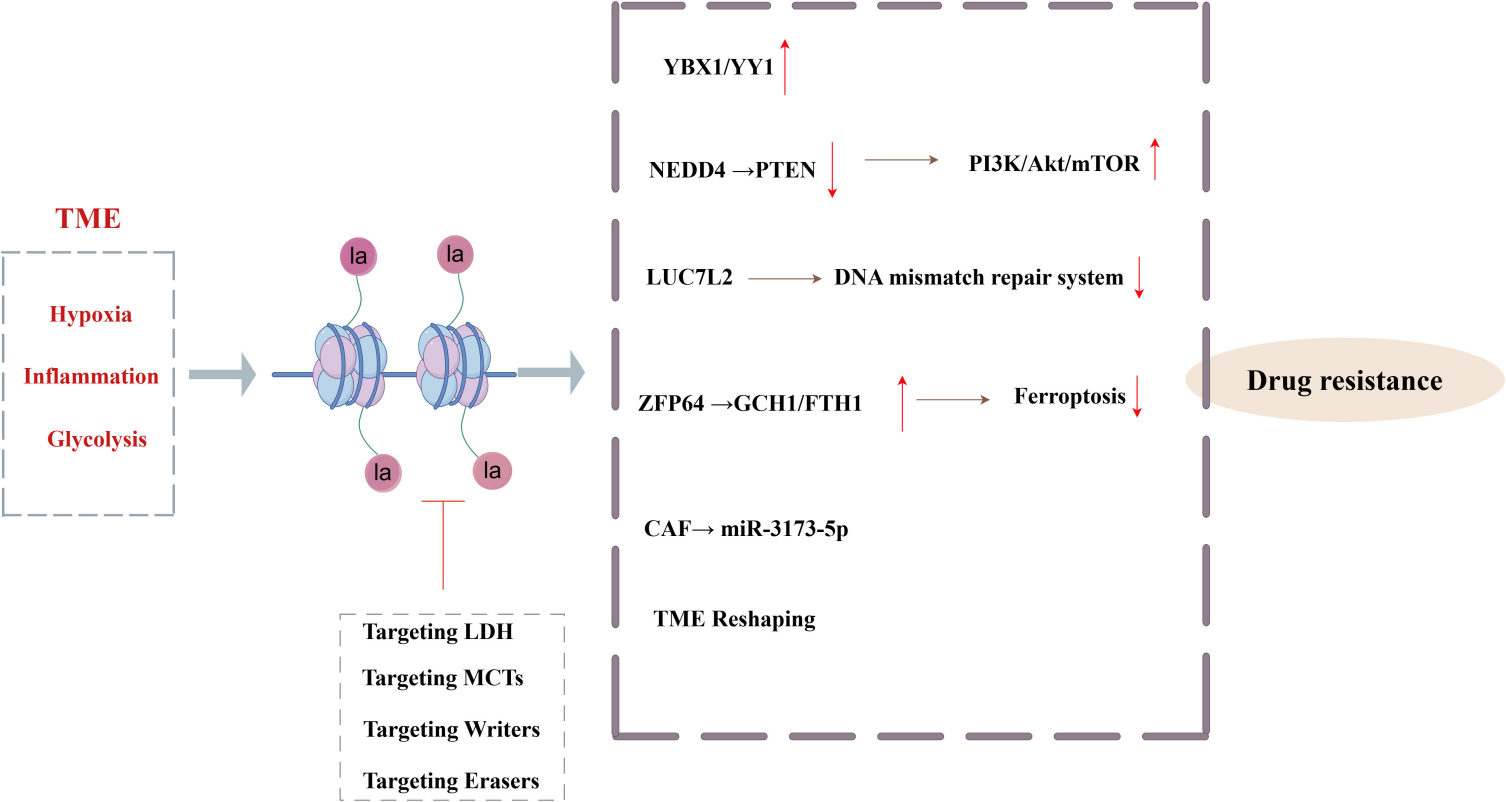
Core mechanisms and targeted interventions of histone lactylation-mediated tumor chemotherapy resistance (By Figdraw). The hypoxia, inflammation and glycolysis signals in TME drive Kla, which, through YBX1\YY1, activates the PI3K/Akt/mTOR signaling pathway, inhibits the mismatch repair system, inhibits ferroptosis, and remodels the TME to mediate tumor resistance.

Chemotherapy is a cornerstone in the treatment of malignant tumors, even though chemoresistance significantly limits its therapeutic efficacy and degrades patient outcomes (55). Extensive studies in recent years have shown that Kla and tumor drug resistance are related. In the TME, Kla acts as a crucial bridge connecting tumor metabolism and epigenetic regulation, converting signals related to inflammation, hypoxia, and glycolysis into multifaceted resistance mechanisms. Kla specifically activates immune checkpoint (including PD-L1), oncogenes, factors linked to the epithelial-mesenchymal transition (EMT), and drug resistance gene transcription programs, all of which completely alter the drug-resistant phenotype of cancer cells. It stimulates angiogenesis, immunological suppression, protective autophagy, and the ability to repair DNA damage (56) (**Figure 3**). Numerous studies have demonstrated how lactate, a post-translational alteration induced by a tumor metabolite, influences the outcomes of chemotherapy by Kla. For example, lactate-mediated interactions between tumor cells and CAFs in gastric cancer increase anlotinib resistance (57). Histone H3K9la causes temozolomide resistance in glioblastoma by triggering the transcriptional repressor LUC7L2, which harms the DNA mismatch repair system (58). Similar to this, H3K18la upregulates the transcription factors Y-box binding protein 1 (YBX1) and Yin Yang 1 (YY1) in bladder cancer, promoting anlotinib resistance and ultimately cisplatin resistance (59). In pancreatic ductal adenocarcinoma, CAFs inhibit ferroptosis by releasing exosomal miR-3173-5p, which leads to gemcitabine resistance (60). According to recent research, histone Kla activates the PI3K/Akt/mTOR signaling pathway and causes multidrug resistance in hepatic cancer by controlling the production of the E3 ubiquitin ligase NEDD4, which in turn increases PTEN ubiquitination and degradation (61). NEDD4 is highly expressed in BC and is associated with a poor prognosis (62, 63), indicating that the Kla-NEDD4-PTEN axis may have regulatory effects in BC resistance, even if this mechanism is still unvalidated in BC. Interestingly, CAFs prevent ferroptosis in BC via increasing ZFP64 through histone Kla, which then promotes the transcription of GCH1 and FTH1. This pathway facilitates the blocking of lipid peroxidation and the depletion of intracellular Fe^2+^ in TNBC, leading to adriamycin resistance (64). Inhibitors of histone delactylase have also shown promise in overcoming the chemoresistance of TNBC. Specifically, METTL3’s delactylation by HDAC2 enhances cisplatin resistance and DNA damage repair. Tucidinostat, an HDAC inhibitor, is a potential therapy option for TNBC that may help restore Kla and lessen chemoresistance (65). In conclusion, focusing on the Kla-mediated regulatory system may present fresh therapeutic approaches and tactical avenues for combating chemotherapy-resistant tumors.

### 4.3 Lactylation regulates the tumor microenvironment in breast cancer

**Figure 4:**
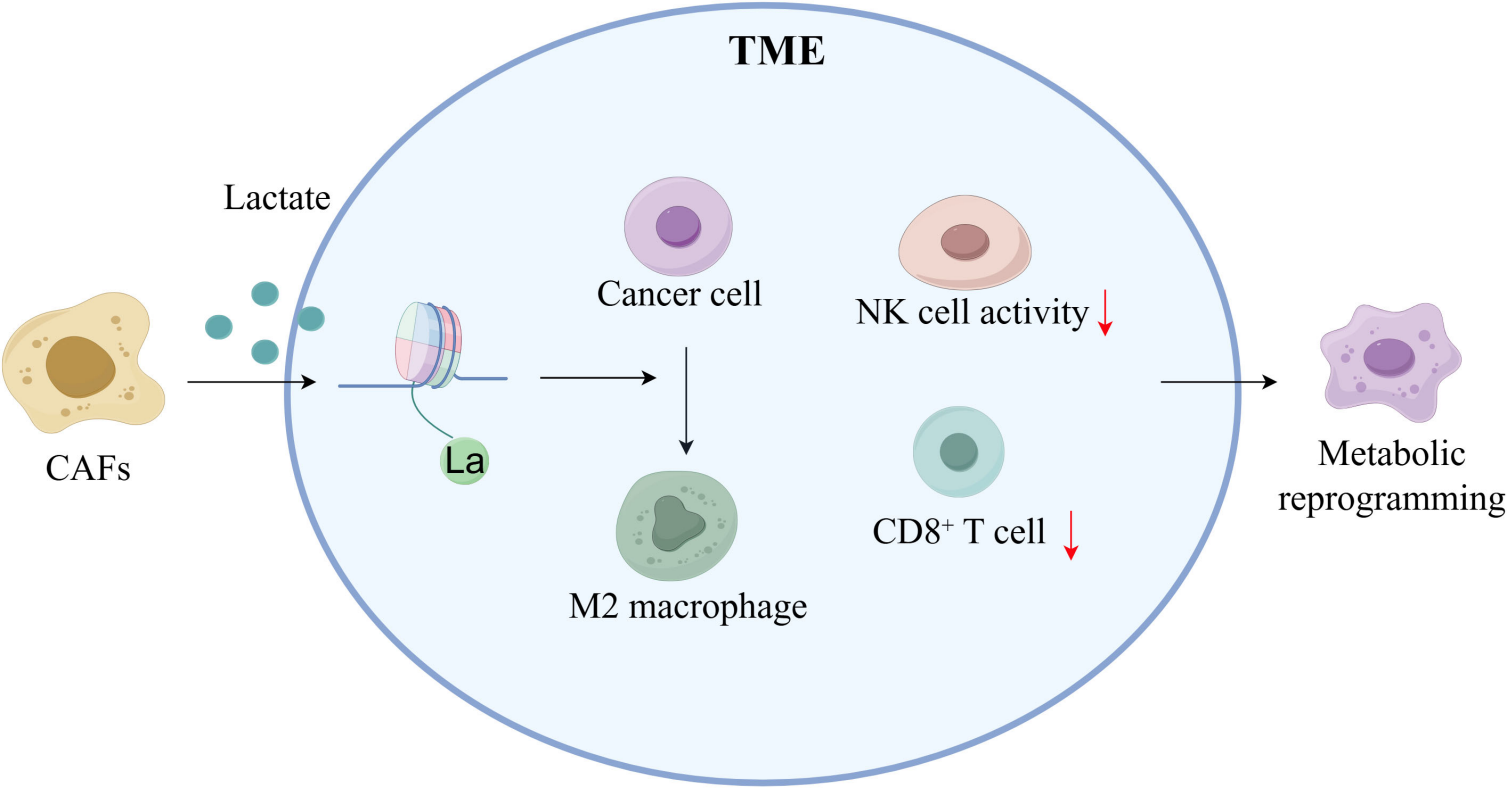
Lactylation regulates the tumor microenvironment in breast cancer (By Figdraw). CAFs elevate lactic acid levels within the TME through secretion, promoting the formation of an acidic microenvironment. Elevated lactic acid induces metabolic reprogramming, activates M2 macrophage polarization, induces CD8^+^ T cell exhaustion, reduces NK cell activity, and promotes tumor cell invasion and immune evasion.

The TME is a complex ecosystem that includes cancer cells, immune and inflammatory cells, CAFs, surrounding mesenchymal tissues, microvasculature, and various cytokines and chemokines (66). Here, the lactate released by CAFs significantly increases the amount of lactate in the TME, causing metabolic reprogramming and contributing to the formation of an acidic environment that promotes tumor growth (67). This lactate-induced metabolic reprogramming promotes tumor growth, invasion, and metastasis. Metabolic reprogramming, which alters cellular energy metabolism to encourage rapid cell proliferation, is a feature of cancer (68). Kla exacerbates the immunosuppressive environment by promoting M2-type macrophage polarization, inhibiting natural killer (NK) cell function, and resulting in CD8(+) T cell exhaustion (**Figure 4**). Additionally, the expression of genes associated with BC, such as NDUFAF6, OVOL1, and SDC1, has been positively correlated with M2 macrophage infiltration, supporting the immunological escape phenotype (69). The positive association between the expression of genes associated with Kla and immune cell infiltration, particularly dendritic cells and T cells, which are significantly elevated in high-risk BC, suggests that Kla may influence the progression of the disease by regulating immune cell recruitment and function (70). Furthermore, hypoxic CAFs increase lactate generation, which raises mitochondrial activity and invasive capacity and facilitates the spread of BC cells, according to Sun et al. (10). Together, these findings demonstrate the importance of further investigation into the connection between lactate metabolism in the TME and immunological regulation. These findings will provide a theoretical basis for developing innovative therapeutic strategies that focus on the metabolic–immune axis in BC.

### 4.4 Lactylation and cancer markers in breast cancer

Despite recent research highlighting Kla’s crucial involvement in cancer metabolism, nothing is known about its potential as a diagnostic for particular tumor types. Numerous studies have started to fill this gap. By upregulating H3K18la and activating LDH, KCNK1 has been demonstrated to facilitate tumor cell proliferation and metastasis in BC (52). Additionally, studies reveal that histone H4K5la is much higher in BC patients than in healthy individuals and is increased in peripheral blood mononuclear cells (PBMCs), indicating its potential as a non-invasive diagnostic biomarker (71). H4K12la levels were shown to be significantly higher in TNBC by Kla proteomics analysis. These levels were negatively correlated with patient overall survival (OS) and positively correlated with the tumor growth marker Ki-67 (65, 66). The protein Kla profile in TNBC was further mapped by Cui et al. (72, 73), who also confirmed that H4K12la is substantially expressed in TNBC tissues and strongly linked to a poor prognosis. According to these results, H4K12la may be a predictive biomarker for TNBC with substantial therapeutic utility. This opens up new possibilities for research into targeted BC therapies and Kla-based therapy approaches.

### 4.5 Lactylation and circadian rhythms in breast cancer

In normal cells, metabolic processes are tightly regulated by circadian rhythms. However, in tumor cells, circadian dysregulation subsequently interacts with cellular metabolism, jointly supporting tumor cell proliferation, survival, invasion, and metastasis (74). Normal cells, tissues, and organs display unique circadian rhythmic patterns in glycolysis, which is regulated in part by the biological clock (75). Numerous investigations show that circadian rhythms are powerful tumor suppressors (76, 77) and that BC is linked to their disruption (78, 79). In BC, lung metastases and an immunosuppressive TME are encouraged by long-term disruption of the circadian cycle, such as jet lag (80). Circadian rhythms control metabolism; mutations in BMAL1 or the lack of PER2 and BMAL1 increase MYC transcription, glucose consumption, and lactate excretion, which speeds up the development of lung cancer (81, 82). Circadian rhythms are also controlled by metabolism, and TME inputs have particular regulatory effects on biological clock function that rely on lactate generated through glycolysis and cytokine production (83). According to research, the acidic TME interferes with the circadian clock by blocking the mTORC1 complex, which lowers the translation of key clock proteins like BMAIL1 when hypoxic conditions and elevated lactate generation are present (84). Thus, we suggest that lactate and circadian rhythms are regulated in both directions: lactate accumulation further interferes with circadian function through inhibition of the Kla-mediated signaling pathway, whereas circadian dysregulation increases lactate generation through increased glycolysis. One important factor in the connection between metabolic malfunction and circadian disruption is lactate, which is both a metabolic waste and a substrate that induces Kla. Subsequent research on the complex interplay between Kla and circadian rhythms may provide fresh insights for creating BC treatment plans that focus on the bidirectional modulation of Kla and timing.

## 5 Therapeutic potential of targeted lactylation in breast cancer

**Figure 5:**
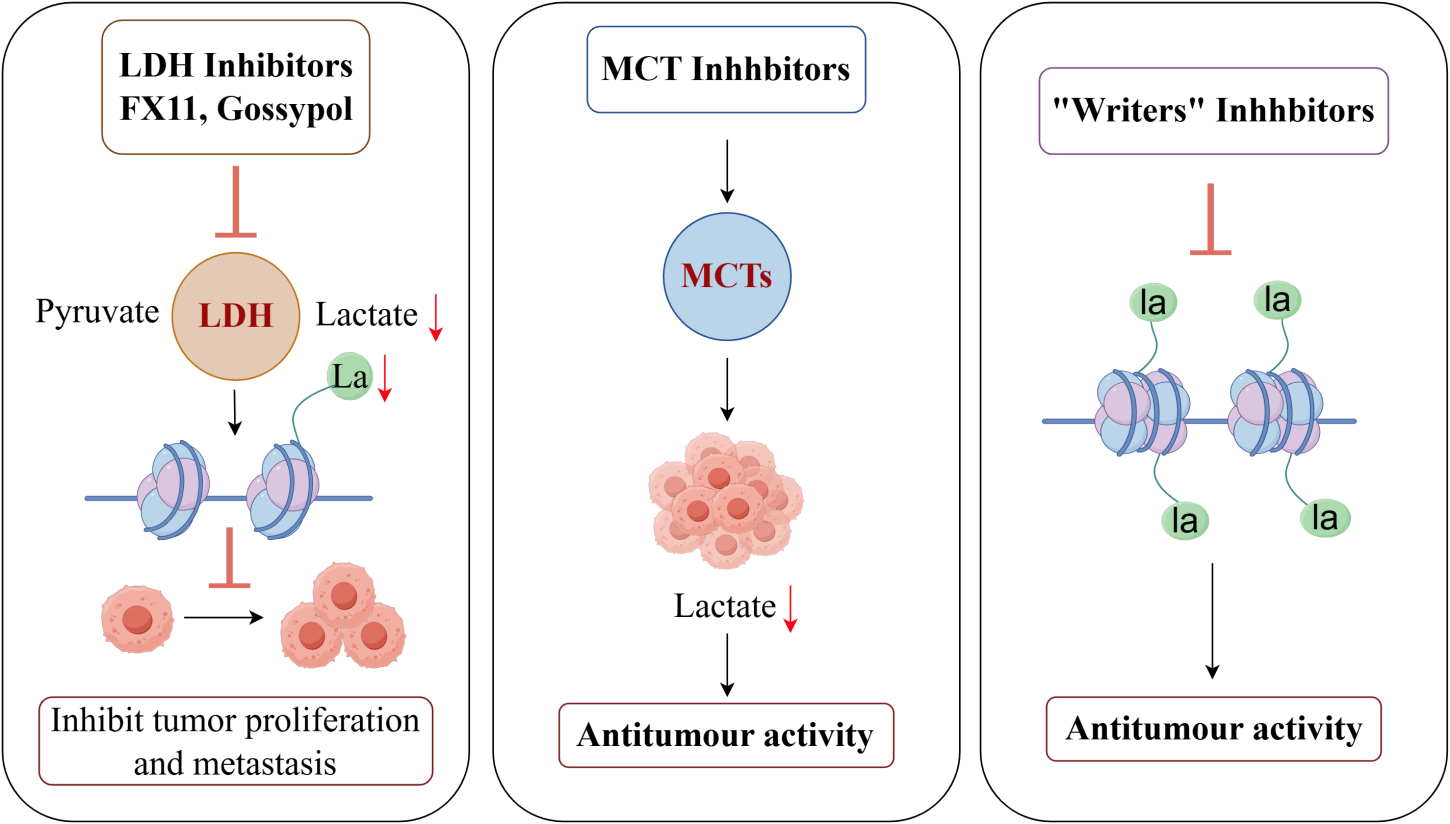
Therapeutic potential of targeted lactylation in breast cancer (By Figdraw). LDH inhibitors such as FX11 and Gossypol significantly reduce intracellular lactate levels and lactylation by blocking the conversion of pyruvate to lactate, thereby inhibiting tumor proliferation and metastasis. MCT1 inhibitors prevent lactate efflux, reducing lactate accumulation in the TME and demonstrating antitumor activity. Lactyl transferase (Writers) inhibitors block the lactylation of histones and non-histones, thereby suppressing the transcription of tumor-associated genes and exhibiting significant antitumor effects.

Targeting this alteration process has become a very attractive anti-cancer treatment because of the crucial function that Kla plays in tumor development. This strategy’s main goal is to upset the lactate metabolic homeostasis inside tumor cells, which is accomplished mainly by three main methods: first, blocking the production of lactate (for example, by targeting lactate dehydrogenase, or LDH) and its transport (for example, by targeting monocarboxylate transporters, or MCTs); Second, “writers” (like lactosyltransferases) and “erasers” (such lactate deacetylases) dynamically regulate Kla itself (85)(**Figure 5**). The creation of particular inhibitors that target these “writers” and “erasers” thus constitutes another crucial avenue. In conclusion, lactate production/transport, writers, and erasers are the three main targets of contemporary Kla-targeted medication development.

### 5.1 Targeting LDH

Pyruvate and lactate are converted in both directions by the tetrameric enzyme LDH. By avoiding OXPHOS, cancer cells primarily use LDHA to redirect pyruvate, a metabolic precursor, toward the pentose phosphate pathway, which promotes the growth of cancer cells (86–89). In the meantime, it has been discovered that LDHB is overexpressed in malignancies of the breast, thyroid, lung, and pancreas and that its presence is strongly linked to a bad prognosis (90–92). By preventing the conversion of pyruvate to lactate, LDH inhibitors like FX11 and Gossypol drastically lower intracellular lactate levels and Kla in TNBC models, hence inhibiting tumor proliferation and metastasis (93, 94). These studies imply that by interfering with the dual pathways of lactate metabolism and Kla, particular inhibitory techniques that target LDH isoforms (e.g., LDHA/LDHB) can be accomplished, hence preventing the progression of tumors. For the creation of metabolic precision treatments for BC, this offers a crucial theoretical foundation. The goal of current research is to create LDH inhibitors that are safer, more selective, and more potent in cells.

### 5.2 Targeting MCTs

A promising approach to cancer treatment is to target MCT proteins, which have been demonstrated to prevent cancer cell motility, invasion, and metastasis (95). There are currently many different types of MCT inhibitors on the market, such as CHC (96–99), organomercurial compounds (100), photothialdehyde benzenesulfonate (100), and second-generation medications with better selectivity, like AR-C155858 (101, 102), which targets MCT1/2, and BAY8002 and SR13800, which target MCT1 (103, 104). In MCT1-positive models of Burkitt’s lymphoma, as well as breast and stomach malignancies, AZD3965 has shown therapeutic efficacy (103, 105). Additionally, in preclinical models of breast and lung malignancies, the MCT1 inhibitor AZD3965 has demonstrated anti-tumor efficacy by reducing lactate buildup in the TME and blocking lactate efflux (106). Research indicates that combining medications that target MCT with other therapy modalities may improve therapeutic results. For example, Li et al. (107)found that the MCT inhibitor Syrosingopine reversed the immunosuppressive TME in a mouse model of BC by upregulating NK cells and the M1 phenotype of tumor-associated macrophages (TAMs) and downregulating the frequency of Treg cells. Furthermore, Ma et al. discovered that lithium carbonate (LC) encouraged the endocytosis of lactate into mitochondria and helped localize MCT1 to the mitochondrial membrane. In CRC, melanoma, and BC, this increased energy production helped restore tumor-responsive CD8(+) T cells and sensitize immunotherapy (108). Therefore, MCT-targeted treatments and immunotherapy may work in concert to increase treatment effectiveness.

### 5.3 Other strategies for targeting lactate

Important regulators of Kla include delactase-like enzymes (Erasers) and lactosyltransferases (Writers). Research has shown that p300 inhibitors, such as A-485 and C646, decrease the transcription of tumor-associated genes by blocking the Kla of histones and non-histone proteins. They also show notable anti-tumor effects in models of triple-negative breast and prostate cancer (109, 110). Furthermore, by lowering lactate content, producing hydrogen peroxide (H2O2), and attracting immune cells, researchers have suggested that lactate oxidase (LOx) can overcome immunosuppression and sensitize immunotherapy (111, 112). The production of pyruvate by LOx-catalyzed lactate consumption triggers CRISPR/Cas9-mediated editing of the signaling-regulatory protein alpha (SIRPα) genomic plasmids. In an *in situ* BC model, LOx inhibits tumor growth by promoting the conversion of M2-type macrophages to M1-type macrophages through the formation of nanoparticles (LPZ: LOx, Cas9/sgSIRPα plasmid, mannose-modified PEG-loaded ZIF-67) in conjunction with these plasmids and metal-organic frameworks (MOFs) (113). In order to counteract immunosuppressive effects in the TME, Zhang et al. also created a metal-phenolic network nanocomplex that contains LOx and the mitochondrial respiration inhibitor atovaquone (ATO). In sonodynamic treatment (SDT) for BC, this nanocomplex demonstrated better pharmacological effects than monotherapy (114). Using alkaline salts to neutralize lactate is another possible targeted treatment approach. In metastatic BC mice models, it has been shown that neutralizing tumor-derived lactate with alkaline salts like sodium bicarbonate (NaHCO_3_) substantially suppresses spontaneous metastasis (115). Moreover, acid-neutralizing calcium carbonate nanoparticles have been used to prevent cellular migration and proliferation in addition to keeping BC cell pH within typical physiological levels (116).

Kla as a novel field formally reported only in 2019, has yet to see any drug enter clinical trials directly as a ‘sre inhibitorT due to several current challenges. Firstly, important Kla enzymes like p300 and HDACs have a wide range of functions and take part in other essential physiological processes including acetylation. The creation of highly selective inhibitors is a big issue because direct inhibition may result in serious side effects (117). Second, it is still challenging to accurately differentiate the functional functions of D-lactate and L-lactate alteration *in vivo*. Lactate is also a typical metabolic byproduct. Inhibiting Kla systemically and arbitrarily runs the danger of upsetting regular cellular metabolism and producing unanticipated negative effects. For upcoming clinical applications, precise drug delivery to tumor locations is still a crucial concern. Indirect approaches that target lactate metabolic pathways are gradually making their way into clinical research, even though direct medicines that target Kla are still in their infancy. As the first medication to target lactate metabolism, AZD3965, an MCT1 inhibitor, has entered Phase I/II clinical trials (NCT01791595) for the treatment of non-Hodgkin lymphoma and advanced solid tumors (118, 119).

Even though some enzymes have been identified as possible therapeutic targets, the intricate interactions between various pathways necessitate a more organized, multitarget, process-directed therapeutic approach that seeks to concurrently interfere with several oncogenic processes in order to reduce the number of potential “escape” mechanisms that tumors may use (120). Therefore, in order to give new theoretical underpinnings and intervention targets for this multi-target strategy, future research must continue to focus on investigating the novel mechanism of Kla in carcinogenesis.

## 6 Challenges and future directions

Lactate has been shown to be an essential signaling molecule within the TME, going beyond its conventional understanding as an energy substrate in the context of cancer metabolism in recent years. In addition to providing energy for the tricarboxylic acid cycle, which supports the energy metabolism of cancer cells, lactate also moves between cells through MCTs, creating a pattern known as “metabolic symbiosis” that encourages metabolic cooperation between various cell populations within the TME. Proteomic advances have also shown that lactate induces a new PTM called Kla. Enzymatic (like p300) and non-enzymatic pathways, which are dynamically controlled by “writers,” “erasers,” and “readers” (like HDAC1–3 and BRD family proteins), are responsible for this alteration. Lactate buildup in tumor cells raises Kla levels, which affects vital functions such as tumor development, metabolic reprogramming, circadian rhythm disruption, and immune control. By affixing lactate groups to lysine residues, histone Kla directly connects metabolic fluctuations with the regulation of gene expression, hence connecting metabolic conditions to epigenetic regulation. By providing a fresh theoretical perspective on tumor plasticity and heterogeneity, this process greatly enhances the various facets of cancer research.

Lactate alteration in BC directly enhances tumor cell proliferation, invasion, and metastatic potential by restructuring cellular metabolic networks (including augmenting glycolysis, suppressing OXPHOS, and creating disruptions in circadian rhythms) and modulating critical signaling pathways. Additionally, Kla may accelerate the course of the disease by upregulating pathways linked to DNA damage repair, which might result in treatment resistance. CAFs use aerobic glycolysis to produce energy quickly inside the TME. They provide substrates for their energy metabolism by moving large volumes of lactate to other cells in the TME via the “lactate shuttle” process. This process causes the TME’s lactate concentrations to rise noticeably, which not only produces an acidic environment that promotes cancer growth but also provides protein Kla with plenty of substrate conditions. The potential of Kla-associated genes as predictive biomarkers for BC is shown by the strong correlation between their expression patterns and immunosuppressive characteristics. Based on the above mechanisms, targeting lactate synthesis and its associated lactate-mediated modifications is anticipated to not only reverse the immunosuppressive milieu but also to elicit various anti-tumor actions by blocking lactate-dependent oncogenic signaling pathways. Consequently, the intervention method targeting lactate offers a significant avenue for the advancement of BC treatment in the future.

Strategies that target Kla-modified regulatory proteins (e.g., HDAC inhibitors) and important nodes of lactate metabolism (e.g., LDHA inhibitor FX11, MCT1/4 inhibitor AZD3965) have demonstrated encouraging anticancer potential. Nevertheless, it is still difficult to evaluate the impact of lactate homeostasis modulation on efficacy and conduct thorough studies into its specificity and safety. The sensitivity and specificity of current Kla detection techniques (such as anti-lactate antibodies) in tissue samples are insufficient. The advancement of their use as diagnostic and prognostic indicators depends on the development of high-precision detection instruments (such as probes tagged with mass spectrometry). The necessity for tumor-selective delivery systems (such nanocarrier-loaded LDHA inhibitors) or dual-target medications (like simultaneous inhibition of LDHA and HDAC) is highlighted by the danger of systemic metabolic disruptions associated with current LDH/MCT-targeting therapies. Furthermore, metabolically focused therapies are less effective due to the TME’s complexity and heterogeneity. Achieving effective medication concentrations at the tumor site is challenging due to this heterogeneity, which is evident throughout different stages of BC, clinical phases, and metabolic differences between distinct cell types within the TME. Lactate inhibitors frequently have low selectivity and substantial off-target effects when used on cancers with phenotypic plasticity, making it more difficult to determine their actual pharmacological mechanisms and therapeutic benefits. Furthermore, the identification of Kla “Erasers” and “Readers” is still lacking, especially in relation to the screening of regulatory proteins specific to BC and the comprehension of their interplay with epigenetic mechanisms. It is yet unknown how Kla varies in time and space among various TME cell subpopulations (such as CAFs, immune cells, and tumor cells) and how it affects immunological checkpoints (such PD-L1). It is necessary to use spatial metabolic imaging and single-cell sequencing to clarify these dynamic networks.

Kla plays a significant role in the occurrence and development of cancer, which will be of great benefit for the design of combined therapies. For instance, combining Kla inhibitors with immune checkpoint inhibitors, standard chemotherapy/targeted therapies, and drugs targeting key glycolytic enzymes may open up new avenues for overcoming drug resistance and immune evasion in cancer treatment. Thus, examining Kla as a key link between malignant phenotypes and metabolic abnormalities advances our knowledge of the pathophysiology of BC and offers a theoretical basis for creating targeted therapeutic approaches that target the metabolic-epigenetic-immune microenvironment. Moving this topic from mechanistic exploration to therapeutic application in the future will require interdisciplinary cooperation and technical innovation.
